# Detection of drug resistance in *Escherichia coli* from calves with diarrhea in the Tongliao region: an analysis of multidrug-resistant strains

**DOI:** 10.3389/fvets.2024.1466690

**Published:** 2024-11-13

**Authors:** Zi Wang, Miao Sun, Shuang Guo, Yongqiang Wang, Linghao Meng, Jinchuan Shi, Chao Geng, Dongxu Han, Xiaomeng Fu, Jiangdong Xue, Hongxia Ma, Kai Liu

**Affiliations:** ^1^College of Animal Science and Technology, Inner Mongolia Minzu University, Tongliao, China; ^2^Inner Mongolia Engineering Technology Research Center for Prevention and Control of Beef Cattle Diseases, Tongliao, China; ^3^Beef Cattle Industry School of Inner Mongolia Autonomous Region, Tongliao, China; ^4^Hinggan League Animal Disease Control Center, Hinggan League, China; ^5^Zhalantun Vocational College, Hulunbeier, China; ^6^Tongliao Vocational College, Tongliao, China; ^7^College of Animal Science and Technology, Jilin Agricultural University, Changchun, China

**Keywords:** *Escherichia coli*, calf diarrhea, drug resistance, antimicrobial resistance genes, whole genome sequencing

## Abstract

**Introduction:**

*Escherichia coli* is a major pathogen responsible for calf diarrhea, which has been exacerbated by the irrational and unscientific use of antimicrobial drugs, leading to significant drug resistance.

**Methods:**

This study focused on the isolation and identification of *E. coli* from calf diarrhea samples in the Tongliao area of China. Isolation was conducted using selective media, Gram staining, and 16S rRNA sequencing. The minimum inhibitory concentration (MIC) of *E. coli* was determined through the microbroth dilution method. Additionally, the presence of antibiotic-resistant genes was detected, and multidrug-resistant strains were selected for whole-genome sequencing (WGS).

**Results:**

The results revealed that all 40 isolated strains of *E. coli* exhibited resistance to sulfadiazine sodium, enrofloxacin, and ciprofloxacin, with 90% of the strains being susceptible to polymyxin B. Notably, strains 11, 23, and 24 demonstrated severe resistance. The detection rates of the antibiotic resistance genes *TEM-1, TEM-206*, *strA*, *strB*, *qacH*, and *blaCTX* were 100%, indicating a high prevalence of these genes. Moreover, the majority of strains carried antibiotic resistance genes consistent with their resistance phenotypes. WGS of strains 11, 23, and 24 revealed genome sizes of 4,897,185 bp, 4,920,234 bp, and 4,912,320 bp, respectively. These strains carried two, one, and two plasmids, respectively. The prediction of antibiotic resistance genes showed a substantial number of these genes within the genomes, with strain 24 harboring the highest number, totaling 77 subspecies containing 88 antibiotic resistance genes.

**Discussion:**

In conclusion, all 40 isolated strains of E. coli from calf diarrhea in this study were multidrug-resistant, exhibiting a broad distribution of antibiotic resistance genes and mobile components. This poses a significant risk of horizontal gene transfer, highlighting the critical situation of antibiotic resistance in this region.

## 1 Introduction

Neonatal calf diarrhea (NCD) presents a formidable challenge to the global livestock industry, significantly hindering sector growth. This disease incurs substantial economic losses due to its high rates of morbidity and mortality, growth retardation in affected animals, and the associated treatment costs, along with severe long-term consequences (1–3). NCD is a leading cause of mortality in calves within their first month of life, with approximately 57% of pre-weaning calves succumbing to diarrhea (4–6). The etiology of NCD is multifactorial, with infections by various pathogens playing a critical role, compounded by factors such as genetics, age, environmental conditions, and nutritional status. The predominant pathogens causing NCD include *Bovine Viral Diarrhea Virus*(BVDV), *Bovine Rotavirus* (BRV), *Bovine Coronavirus* (BCoV), *E. coli*, *Salmonella*, *Clostridium perfringens*, *Cryptosporidium*, and *Eimeriidae. E. coli*, in particular, is highly contagious and can cause severe diarrhea and septicemia in calves, with higher morbidity and mortality rates observed in younger animals (7, 8).

Pathogenic *E. coli* is typically categorized into intestinal and extraintestinal pathogenic strains. The intestinal pathogenic strains are further divided into six types: Enterotoxigenic *E. coli* (ETEC), Enterohaemorrhagic *E. coli* (EHEC)/Shiga toxin-producing *E. coli* (STEC), Enteropathogenic *E. coli* (EPEC), Enteroinvasive *E. coli* (EIEC), Enteroaggregative *E. coli* (EAEC), and Diffusely adherent *E. coli* (DAEC). Among these, ETEC is the primary strain responsible for diarrhea in calves (9–12). ETEC’s pathogenic mechanism involves bacterial adhesion and colonization of the small intestinal epithelial cells via fimbriae, followed by the transfer of small peptides or secretion of enterotoxins that trigger a cascade of intracellular signals. This leads to a rapid loss of electrolytes from the intracellular environment to the intestinal lumen, resulting in watery diarrhea in calves (13–15).

In veterinary practice, while antibiotics are often used to treat bacterial NCD, the application of antibiotic therapy for ETEC infections remains controversial. In this respect, most authors only justify its use in cases that are evolving toward systemic disease, both for the prevention of bacteriemia and to reduce the number of ETEC in the gut. However, antimicrobials are commonly used by veterinary surgeons for teating NCD, even in the absence of demonstrated ETEC involvement. This increased use of antibiotics has accelerated the emergence and spread of bacterial resistance, giving rise to multidrug-resistant (MDR) strains that pose a significant threat to global public health (16, 17). The emergence of bacterial resistance has promoted the development of colostrums and probiotics. Several studies have suggested the benefits of using bovine colostrum and probiotics as prophylactics to prevent diarrhea in calves by the administration of colostrum and probiotics after the first day of life. However, there is a lack of evidence supporting the use of maternally derived bovine colostrum and colostrum replacements as a therapy for diarrhea in calves (18). *E. coli*, serving as a potential source, intermediate carrier, and crucial reservoir of antibiotic resistance genes (ARGs), plays a pivotal role in the dissemination of bacterial resistance (19). Horizontal gene transfer (HGT) via plasmid-mediated conjugation is one of the primary mechanisms for the spread of ARGs (20, 21). HGT is facilitated by several well-known mechanisms including transduction, transformation, and conjugation, with conjugation being the most significant.

HGT is mediated by mobile genetic elements (MGEs), which are DNA molecules capable of moving between replicons (intracellular mobility) or between bacterial cells (intercellular mobility). MGEs include conjugative DNA elements (plasmids and integrative and conjugative elements [ICEs]), transposable DNA elements (transposons and integrons), and bacteriophages. These elements carry various genes, including those for antimicrobial and metal resistance, virulence, and catabolism. Recently, antimicrobial-resistant (AMR) bacteria have emerged as a critical public health threat. The spread of ARGs through HGT-associated MGEs, coupled with the role of MGEs in promoting the diversification of AMR bacteria, exacerbates this issue (22–24). This study aimed to understand the drug resistance of *E. coli* causing diarrhea in calves in the Tongliao area of China. A preliminary assessment was conducted through the isolation and identification of bacteria, drug resistance analysis, detection of drug resistance genes, and analysis of mobile components. The findings provide guidance for the clinical treatment of calf diarrhea caused by *E. coli* in the Tongliao area. They also establish a research basis for further study and control of the spread of bacterial resistance.

## 2 Materials and methods

### 2.1 Sample collection and strain isolation

Between May 2021 and May 2022, fecal samples from 50 calves with diarrhea symptoms were collected from cattle farms in Tongliao City, Inner Mongolia. The sample group consisted of 28 female and 22 male Simmental calves within 1 month of age. None of the sampled calves had been treated with antibiotics. The samples were immediately streaked onto *E. coli* Chromogenic Medium and incubated at 37°C for 18 h. This process was repeated two to three times to ensure purity. Single colonies were obtained by isolating and purifying differently colored colonies. The isolated strains were identified by Gram staining and 16S rRNA sequencing. Genomic DNA was extracted from the bacteria using a previously described protocol, and the 16S rRNA gene was amplified using universal polymerase chain reaction (PCR) primers (Table 1). The PCR products were sequenced at Comate Bioscience Co., Ltd. (Jilin, China) and analyzed using BLASTN on the National Center for Biotechnology Information (NCBI) website (25). The *E. coli* ATCC 25922 quality control strain was kindly provided by the Laboratory of Pharmacology and Toxicology, School of Animal Medicine, Jilin Agricultural University.

**Table 1:** Primer sequences.

Primer name	Primer sequence (5′ → 3′)	The length of the amplification/bp
16S-F	AGAGTTTGATCCTGGCTCAG	1,500
16S-R	GGTTACCTTGTTACGACTT	
F4-F	TGAATGACCTGACCAATGGTGGAACC	484
F4-R	GCGTTTACTCTTTGAATCTGTCCGAG	
F5-F	GCGACTACCAATGCTTCTGCGAATAC	230
F5-R	GAACCAGACCAGTCAATACGAGCA	
F6-F	GCCAGTCTATGCCAAGTGGATACTTC	391
F6-R	GTTTGTATCAGGATTCCCTGTGGTGG	
F18-F	TGGCACTGTAGGAGATACCATTCAGC	230
F18-R	GGTTTGACCACCTTTCAGTTGAGCAG	
F41-F	TTAGCAGCGAAGATGAGTGATGGG	515
F41-R	GTACTACCTGCAGAAACACCAGATCC	

### 2.2 Detection of *Escherichia coli* fimbria adhesin

PCR was used to detect the fimbria adhesin gene of *E. coli* isolates. The primer sequences and reaction conditions are shown in Table 1. The PCR products were detected by 1.3% agarose gel electrophoresis.

### 2.3 Drug sensitivity testing

Antimicrobial susceptibility was tested using the broth microdilution method according to Clinical and Laboratory Standards Institute (CLSI) guidelines (26). Minimum inhibitory concentrations (MIC) of each antibiotic were classified as resistant (R), intermediate (I), or susceptible (S) based on CLSI breakpoints, or the National Antibiotic Resistance Monitoring System (NARMS) breakpoints for intestinal bacteria when CLSI breakpoints were unavailable. *E. coli* isolates resistant to three or more antibacterial agents were considered multidrug-resistant (MDR) (27). Tests were conducted in triplicate for each strain, with *E. coli* ATCC 25922 as the quality control strain.

### 2.4 Detection of resistance genes

Primer 5.0 software was used to design primers for the resistance genes. The primer sequences for *E. coli* antibiotic resistance genes are listed in Supplementary Table S1. Extraction of *E. coli* genomic DNA and detection of drug resistance genes were carried out as previously described (28).

### 2.5 Whole genome sequencing of multidrug-resistant strains

The selection of severely drug-resistant strains for whole-genome sequencing was based on drug susceptibility testing. Whole-genome sequencing was conducted on the Nanopore sequencing platform by Biomarker Technologies Corporation (Qingdao, China). High-quality genomic DNA was extracted, and its concentration, purity, and integrity were assessed using a NanoDrop spectrophotometer and Qubit fluorometer (Thermo Fisher, Waltham, MA, United States). Large DNA fragments were filtered using the BluePippin system. A library was prepared using the Oxford Nanopore Technologies (ONT) Template Prep Kit (SQK-LSK109; Oxford, United Kingdom) and the NEB Next FFPE DNA Repair Mix Kit (Ipswich, MA, United States). The high-quality library was sequenced on the ONT PromethION platform, yielding raw sequencing data (29). Filtered subreads were assembled using Canu v1.5 software (30). The assembly results were corrected by Racon v3.4.3 software using three generations of subreads, with further error correction performed using Pilon v1.22 software with second-generation data to obtain a more accurate genome for subsequent analysis.

### 2.6 Data analysis

Gene prediction was conducted using Prodigal v2.6.3 software (31). Gene sequences were cross-referenced with the Gene Ontology (GO) and Kyoto Encyclopedia of Genes and Genomes (KEGG) functional databases for annotation (32, 33). Drug resistance genes were identified by comparing sequences with the antibiotic resistance database using ResFinder software (34–36). Plasmid typing was predicted using PlasmidFinder 2.1 software (36, 37). MobileElementFinder software was employed to predict mobile elements within the genomes (38).

## 3 Results

### 3.1 Identification of *Escherichia coli*

A total of 40 *E. coli* strains were isolated from 50 fecal samples. The isolates showed blue-green colonies on *E. coli* Chromogenic Medium and specific bands close to the size of the target fragment 16Sr RNA (1,500 bp) were amplified in all test samples. The sequencing results, compared via BLAST on the NCBI database, confirmed the isolates were *E. coli*.

### 3.2 Detection of *Escherichia coli* fimbria adhesin

**Figure 1:**
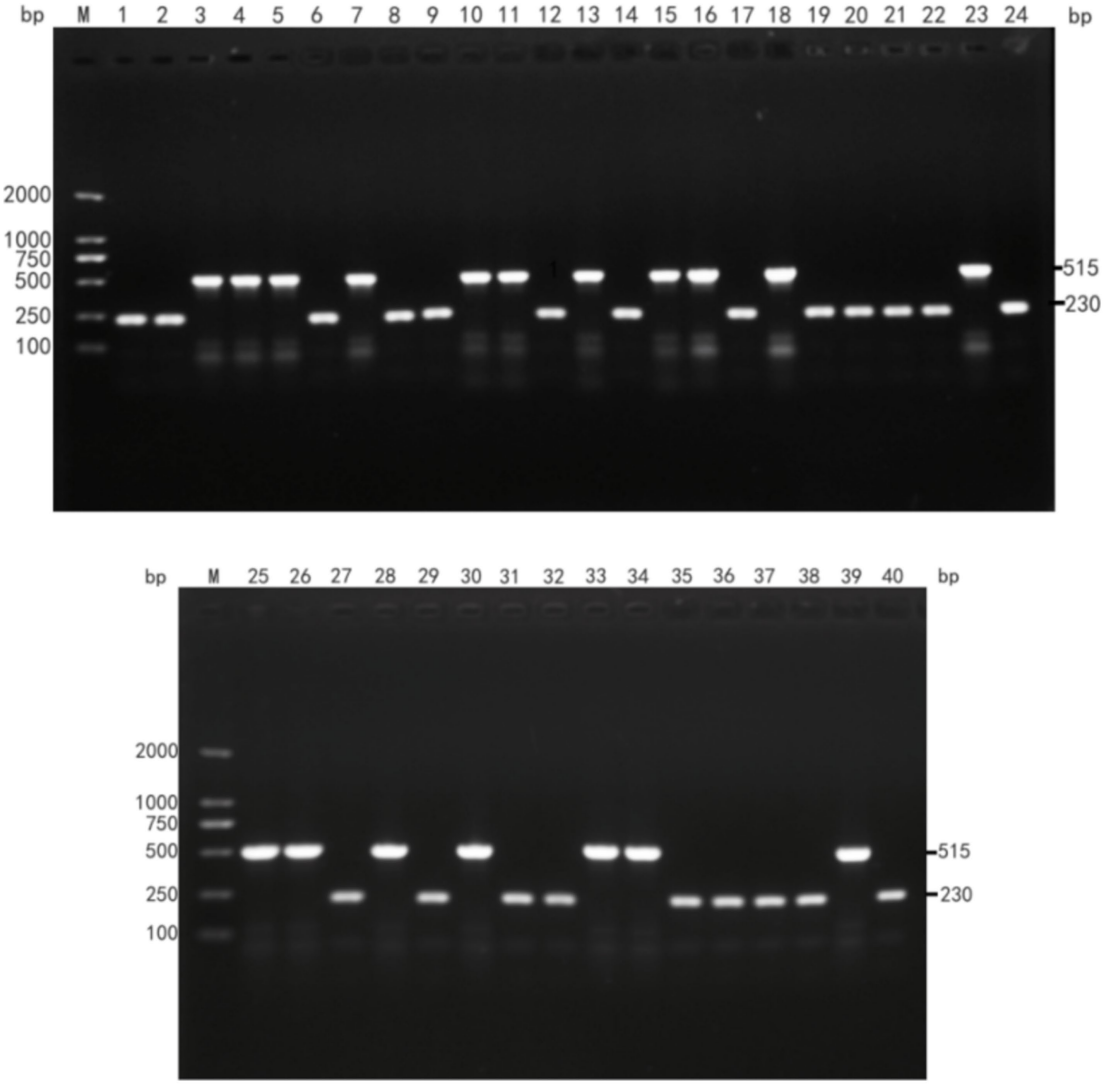
Detection results of *E. coli* fimbria adhesin. (M: DL2000DNA Maeker; 1–40 PCR amplification product of *E. coli* fimbria adhesin).

The fimbria adhesin gene in the 40 *E. coli* genomes was detected using PCR. The detection rates for the F5 gene and the F41 gene were 55% (22/40) and 45%, respectively, while the F4, F6, and F18 genes were not detected. The PCR results for the fimbria adhesin gene are shown in Figure 1.

### 3.3 Drug sensitivity tests

The results of drug sensitivity tests for the 40 *E. coli* isolates are presented in Table 2. The highest resistance rates were observed for sulfadiazine sodium, enrofloxacin, and ciprofloxacin at 100%. In contrast, polymyxin B showed the lowest resistance rate at 10%. *E. coli* strain 24 exhibited resistance to all 11 antibiotics tested, while strains 11 and 23 also demonstrated high resistance. Therefore, these three strains were selected for whole-genome sequencing.

**Table 2:** Results of *E. coli* drug susceptibility testing.

Serial number	Ofloxacin	Kanamycin	Doxycycline	Cephalothin	Tetracycline	Ciprofloxacin	Serial number	Ofloxacin	Kanamycin	Doxycycline	Cephalothin	Tetracycline	Ciprofloxacin
ATCC 25,922	0.5/S	1/S	0.25/S	0.125/S	0.5/S	0.25/S	ATCC 25,922	0.5/S	1/S	0.25/S	0.125/S	0.5/S	0.25/S
1	8/R	32/I	8/I	1/S	4/S	4/R	21	64/R	32/I	>512/R	>512/R	64/R	128/R
2	8/R	256/R	8/I	>512/R	16/R	128/R	22	64/R	64/R	>512/R	>512/R	16/R	128/R
3	16/R	32/I	8/I	512/R	1/S	256/R	23	32/R	16/S	4/S	0.25/S	>512/R	128/R
4	8/R	32/I	4/S	512/R	16/R	128/R	24	64/R	512/R	128/R	>512/R	32/R	128/R
5	8/R	32/I	8/I	512/R	16/R	8/R	25	64/R	512/R	4/S	>512/R	16/R	128/R
6	4/I	32/I	16/R	0.5/S	8/I	2/R	26	64/R	32/I	4/S	512/R	32/R	128/R
7	8/R	64/R	16/R	512/R	16/R	4/R	27	32/R	128/R	1/S	512/R	32/R	128/R
8	8/R	32/I	8/I	1/S	8/I	128/R	28	32/R	64/R	32/R	0.5/S	16/R	128/R
9	8/R	64/R	16/R	512/R	8/I	128/R	29	8/R	32/I	8/I	512/R	16/R	128/R
10	4/I	16/S	8/I	0.25/S	2/S	2/R	30	32/R	16/S	512/R	512/R	32/R	128/R
11	16/R	256/R	8/I	512/R	8/I	128/R	31	32/R	64/R	>512/R	>512/R	32/R	128/R
12	16/R	64/R	32/R	0.25/S	16/R	128/R	32	64/R	16/S	256/R	512/R	16/R	64/R
13	8/R	32/I	0.25/S	512/R	32/R	128/R	33	16/R	16/S	128/R	512/R	64/R	128/R
14	8/R	16/S	4/S	>512/R	128/R	128/R	34	32/R	32/I	4/S	512/R	32/R	128/R
15	64/R	16/S	128/R	512/R	8/I	128/R	35	16/R	16/S	4/S	>512/R	128/R	128/R
16	32/R	16/S	>512/R	512/R	32/R	128/R	36	8/R	16/S	0.5/S	512/R	32/R	128/R
17	16/R	32/I	128/R	512/R	32/R	32/R	37	16/R	32/I	128/R	512/R	64/R	64/R
18	16/R	16/S	64/R	512/R	32/R	128/R	38	16/R	64/R	16/R	>512/R	4/S	128/R
19	16/R	32/I	16/R	>512/R	8/I	128/R	39	64/R	32/I	>512/R	512/R	4/S	128/R
20	64/R	32/I	>512/R	512/R	4/S	128/R	40	64/R	256/R	8/I	>512/R	16/R	128/R

Serial number	Sulfadiazine sodium	Enrofloxacin	Gentamycin	Polymyxin B	Florfenicol	Serial number	Sulfadiazine sodium	Enrofloxacin	Gentamycin	Polymyxin B	Florfenicol
ATCC 25,922	32/S	0.25/S	0.5/S	0.06/S	0.25/S	ATCC 25,922	32/S	0.25/S	0.5/S	0.06/S	0.25/S
1	>512/R	8/R	8/I	0.125/S	2/S	21	>512/R	128/R	>512/R	0.25/S	128/R
2	>512/R	16/R	2/S	0.25/S	8/R	22	>512/R	128/R	>512/R	0.25/S	4/I
3	>512/R	32/R	>512/R	0.125/S	16/R	23	>512/R	256/R	>512/R	0.25/S	>512/R
4	>512/R	8/R	>512/R	0.125/S	16/R	24	>512/R	128/R	>512/R	16/R	32/R
5	>512/R	8/R	>512/R	0.25/S	32/R	25	>512/R	128/R	>512/R	8/R	16/R
6	>512/R	4/R	16/R	0.25/S	0.5/S	26	>512/R	128/R	>512/R	0.125/S	64/R
7	>512/R	8/R	>512/R	0.25/S	32/R	27	>512/R	128/R	>512/R	0.125/S	128/R
8	>512/R	4/R	>512/R	0.125/S	16/R	28	>512/R	128/R	>512/R	0.125/S	2/S
9	>512/R	16/R	>512/R	16/R	32/R	29	>512/R	128/R	>512/R	0.125/S	16/R
10	>512/R	4/R	16/R	0.125/S	0.5/S	30	>512/R	32/R	>512/R	0.125/S	16/R
11	>512/R	32/R	>512/R	0.25/S	8/R	31	>512/R	128/R	>512/R	0.125/S	4/I
12	>512/R	128/R	>512/R	0.125/S	1/S	32	>512/R	128/R	>512/R	0.25/S	16/R
13	>512/R	8/R	>512/R	0.25/S	2/S	33	>512/R	128/R	>512/R	0.125/S	4/I
14	>512/R	16/R	>512/R	0.25/S	>512/R	34	>512/R	128/R	>512/R	0.125/S	32/R
15	>512/R	128/R	>512/R	0.25/S	16/R	35	>512/R	8/R	>512/R	0.06/S	512/R
16	>512/R	64/R	>512/R	0.25/S	16/R	36	>512/R	8/R	>512/R	0.125/S	2/S
17	>512/R	32/R	>512/R	0.25/S	8/R	37	>512/R	32/R	>512/R	0.125/S	4/I
18	>512/R	128/R	>512/R	0.125/S	2/S	38	>512/R	128/R	>512/R	0.125/S	16/R
19	>512/R	32/R	>512/R	0.125/S	16/R	39	>512/R	128/R	>512/R	0.125/S	8/R
20	>512/R	128/R	>512/R	0.25/S	8/R	40	>512/R	128/R	128/R	4/R	16/R

### 3.4 Detection of resistance genes in *Escherichia coli*

The detection results of AMR genes are shown in Table 3. Resistance genes for 25 antibiotics were identified. The highest detection rates were for *β*-lactam antibiotic resistance genes *TEM-1*, *TEM-206*, and *blaCTX*; aminoglycoside resistance genes *strA* and *strB*; and the quaternary amine compound efflux pump gene *qacH*, all at 100%. The lowest detection rate was for the phenylpropanol resistance gene *catI* at 12.5%. The aminoglycoside resistance gene *aadA5* and the sulfonamide resistance gene *sul1* had detection rates above 50%. This indicates that the predominant resistance genes in *E. coli* from calf diarrhea in Tongliao are *qacH*, *strA*, *strB*, *TEM-1*, *TEM-206*, and *blaCTX*. Among the 40 *E. coli* strains, 24 carried the highest number of resistance genes, accounting for 84.62% of the total detected resistance genes. Most resistance genes correlated with their resistance phenotype.

**Table 3:** Results of 40 strains of *E. coli* drug resistance gene detection.

Serial number	Drug-resistance gene	Number of detections	Detection rate (%)	Serial number	Drug-resistance gene	Number of detections	Detection rate (%)
1	aphA1	24	60	14	floR	35	87.5
2	aadA25	26	65	15	mprF	21	52.5
3	aadA5	9	22.5	16	Int1	34	85
4	aadA	33	82.5	17	qnrS	34	85
5	aadA2	31	77.5	18	sul1	19	47.5
6	aadA17	28	70	19	sul2	28	70
7	catI	5	12.5	20	qacH	40	100
8	CTX-M-55	28	70	21	strA	40	100
9	tetA	35	87.5	22	strB	40	100
10	tetD	23	57.5	23	TEM-1	40	100
11	tetR	25	62.5	24	TEM-206	40	100
12	AAC(3)-IIa	23	57.5	25	blaCTX	40	100
13	cmlA6	33	82.5				

### 3.5 Whole genome sequencing analysis of multi-drug resistant strains

#### 3.5.1 Genome assembly statistics

**Figure 2:**
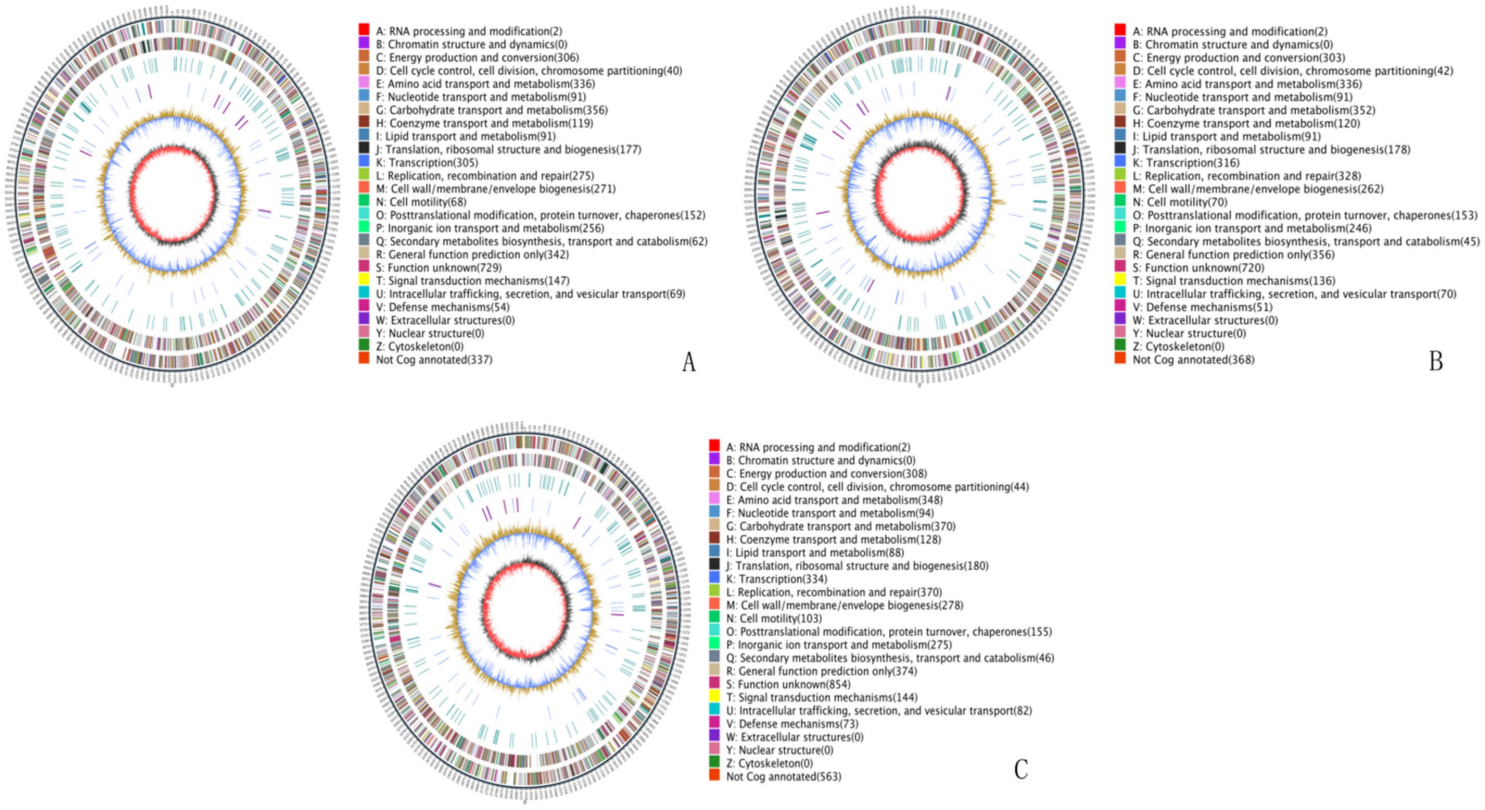
Whole genome maps. **(A)** Whole genome map of strain 11. **(B)** Whole genome map of strain 23. **(C)** Whole genome map of strain 24. The outermost circle is the genome size indication, each scale is 5 kb; the second and third circles are the genes on the positive and negative strands of the genome, respectively, with different colors representing different COG functional classifications; the fourth circle is the repetitive sequences; the fifth circle is the tRNAs and rRNAs, with tRNAs in blue and rRNAs in purple; the sixth circle is the GC-content, and the light yellow portion indicates that the GC-content of the region is higher than the average GC content of the genome, the higher the peak the greater the difference with the average GC content, and the blue part indicates that the GC content of the region is lower than the average GC content of the genome; the innermost circle is the GC-skew, the dark gray represents the region where the G content is greater than the C, and the red represents the region where the C content is greater than the G.

Whole genome sequencing of the three multidrug-resistant strains revealed the following genome lengths: strain 24 had 4,912,320 bp with two plasmids and a G + C content of 50.31%; strain 11 had 4,897,185 bp with two plasmids and a G + C content of 50.68%; and strain 23 had 4,920,234 bp with one plasmid and a G + C content of 50.62%. All sequences have been deposited in the NCBI Sequence Read Archive and are publicly accessible under the accession numbers CP157955-CP157957, CP103295-CP103297 and CP157958-CP157989, respectively. The circular whole genome maps of the three *E. coli* strains are shown in Figure 2. Comparative analysis indicated that strains 11 and 24 showed significant advantages in amino acid transport and metabolism, energy production and conversion, and DNA replication, recombination, and repair. Strain 24 had superior capabilities in energy production and conversion, amino acid transport and metabolism, and carbohydrate transport and metabolism compared to strains 11 and 23, but was less efficient in lipid transport and metabolism, biosynthesis of secondary metabolites, transport and catabolism, and signal transduction mechanisms.

#### 3.5.2 Gene ontology and KEGG function annotation

The genomic sequences of the three *E. coli* strains were annotated for KEGG metabolic pathways. The results showed significant gene involvement in metabolic pathways, particularly in amino acid biosynthesis and carbon metabolism. GO functional annotation indicated that most genes were related to biological processes, especially metabolic processes, cellular processes, and single-organism processes, which are crucial to the strains’ life activities. Strain 24 had more genes related to catalytic activity, metabolic processes, cell membranes, and cell membrane components compared to strains 11 and 23 (see Supplementary Figure S1).

#### 3.5.3 Analysis of resistance genes

Whole genome sequencing of *E. coli* strains 11, 23, and 24 revealed 68, 63, and 77 isoform types of resistance genes, respectively, with total counts of 71, 78, and 88 resistance genes. Chromosomal genomes of strains 11, 23, and 24 carried 51, 57, and 58 types of resistance genes, with counts of 53, 72, and 59, respectively. Plasmid genomes carried 17, 6, and 21 types of resistance genes, with counts of 18, 6, and 29, respectively (see Supplementary Tables S2–S4). Resistance genes in the chromosomal genomes of all three strains mediated resistance to quinolones, aminoglycosides, tetracyclines, macrolides, *β*-lactams, and lincosamide antibiotics. The primary mechanisms included antibiotic efflux, modification-induced inactivation, target site substitution, and alteration of antibiotic targets.

#### 3.5.4 Analysis of mobile components

**Figure 3:**
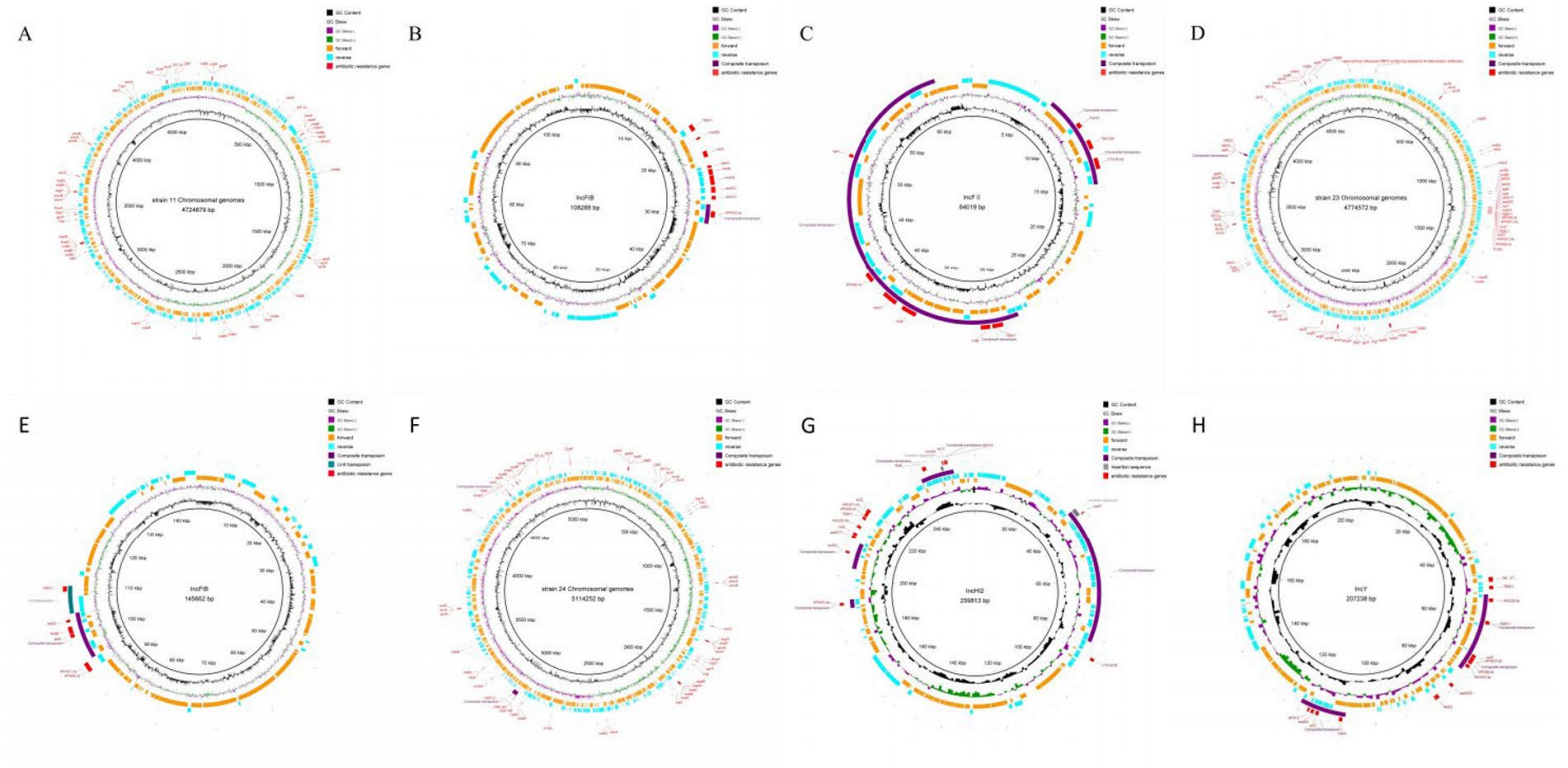
Analysis result of mobile components. **(A)** Result of mobile components analysis of strain 11 Chromosomal genomes. **(B)** Result of mobile components analysis of strain 11 plasmid 1. **(C)** Result of mobile components analysis of strain 11 plasmid 2. **(D)** Result of mobile components analysis of strain 23 Chromosomal genomes. **(E)** Result of mobile components analysis of strain 23 plasmid 1. **(F)** Result of mobile components analysis of strain 24 Chromosomal genomes. **(G)** Result of mobile components analysis of strain 24 plasmid 1, **(H)** Result of mobile components analysis of strain 24 plasmid 2.

Mobile elements in the genomes were analyzed using Mobile Element Finder. The analysis predicted that mobile elements in the genomes of the three *E. coli* strains mainly existed as transposons and insertion sequences, with numerous reverse repetitive sequences (Figure 3). The mobile elements carrying drug resistance genes were primarily composite transposons, mostly from the IS6 family. Only the insertion sequence in plasmid 1 of strain 24 carried a drug resistance gene. Strain 11 contained a compound transposon (TN4352) with no predicted family, while strain 23 had a single transposon (Tn2) with no predicted family (see Supplementary Tables S5–S7).

## 4 Discussion

Calf diarrhea is prevalent in many farms worldwide, characterized by rapid disease progression and high mortality, leading to significant economic losses for the global farming industry (39). Reports indicate that 39% of calves in the United States suffer from diarrhea (40), with similarly high rates observed in Korea, where 53.4% of calf deaths are attributed to this condition (41). ETEC represents a major cause of diarrhea in calves, as well as an important zoonotic pathogen (42). Diarrhea remains a leading cause of morbidity and mortality throughout the world, especially in developing countries, with ETEC being responsible for hundreds of millions of cases of childhood diarrhea each year, accounting for between 1.6 and 2.5 million deaths (43). Since diarrheic calves are a major source of ETEC transmission to humans, effective prevention and treatment of calf diarrhea remain a significant global challenge (44, 45). Antibiotics have traditionally been effective in managing this disease; however, the extensive and often inappropriate use of antibiotics has led to the emergence of antibiotic-resistant *E. coli* strains, posing a severe public health problem globally.

As the national core of beef cattle producing area of China, Tongliao has a large number of beef and the diarrhea caused by *E. coli* is frequently occurring. However, there are few studies on the drug resistance of *E. coli* in this area. In this study, drug sensitivity testing revealed that the 40 *E. coli* isolates from calves with diarrhea exhibited varying degrees of resistance to 11 antibiotics. Sodium sulfadiazine, enrofloxacin, and ciprofloxacin showed the highest resistance rates at 100%, followed by gentamicin, ofloxacin, and thiramycin with resistance rates exceeding 80%. Multidrug resistance was also prevalent. Previous studies, such as those by Yassin et al. and Jia et al., have reported high resistance rates to tetracycline, ciprofloxacin, enrofloxacin, and gentamicin in *E. coli* isolates from different regions in China (46, 47). Similarly, He et al. reported high resistance rates in the Xinjiang region (48). Comparatively, Algammal et al. found lower resistance rates in Egyptian isolates, while Khawaskar et al. reported varied resistance in Indian isolates (11, 15). These variations highlight the regional differences in antibiotic resistance, likely influenced by local therapeutic practices. The high resistance rates observed in this study underscore the severe antibiotic resistance in bovine *E. coli* in Tongliao, China. Although the sample size of strains in our study is relatively small, but in the follow-up study, we will continuous attention to the resistance of *E. coli* strains isolate from calf diarrhea in Tongliao.

Polymyxin B has become a last-resort antibiotic for treating Gram-negative multidrug-resistant bacterial infections (49). In this study, the *E. coli* isolates were generally sensitive to polymyxin B, although 10% showed resistance, emphasizing the need for judicious antibiotic use to prevent further resistance development. The detection of resistance genes revealed that *β*-lactam resistance genes (*blaCTX*, *TEM-1*, *TEM-206*), aminoglycoside resistance genes (*strA*, *strB*), and the quaternary amine compound efflux pump gene (*qacH*) were prevalent, with a 100% detection rate. This high prevalence of resistance genes, coupled with the presence of multiple resistance genes in individual strains, correlates with their resistance phenotypes and indicates severe resistance issues in the Tongliao area.

Whole genome sequencing (WGS) has been increasingly used to analyze the genetic information of drug-resistant strains and the transfer mechanisms of resistance genes (50–52). WGS of three multidrug-resistant *E. coli* strains in this study revealed significant genetic insights. Strains 11, 23, and 24 carried two, one, and two plasmids, respectively. GO functional annotation showed an abundance of genes related to metabolic processes, cellular processes, and single-organism processes. Strain 24, in particular, had more genes associated with catalytic activity, metabolic processes, and cell membrane components, suggesting a role in antibiotic efflux and reduced absorption. KEGG pathway analysis indicated significant enrichment of genes involved in amino acid biosynthesis and carbon metabolism, aligning with the GO annotation results. Previous studies have shown that *E. coli* can develop antibiotic resistance through various mechanisms, with antibiotic resistance mediated by efflux pumps, biofilm formation, and enzymatic modification of antibiotics, among other mechanisms (23, 47, 53). The three *E. coli* strains in this study exhibited multiple resistance mechanisms, with antibiotic efflux being predominant, especially in strain 24, which carried more resistance genes on plasmids compared to strains 11 and 23.

Previous studies have shown that *E. coli* can develop antibiotic resistance through various mechanisms, including efflux pumps, biofilm formation, and enzymatic modification of antibiotics (23, 47, 53). As predicted by the CARD database, the three *E. coli* strains in this study exhibited multiple resistance mechanisms, with antibiotic efflux being the predominant mechanism, especially in strain 24, which carried more resistance genes on plasmids compared to strains 11 and 23. Insertion sequences, the simplest mobile elements in bacterial genomes, play a crucial role in the transfer and dissemination of antibiotic resistance genes (54). Studies have shown that composite transposons, formed by identical or related insertion sequence elements, can facilitate the movement of resistance genes, promoting their spread (55). This study integrated the results of whole-genome sequencing with prediction of mobile genetic elements (MGEs) to analyze the genomes of three *E. coli* strains. It was found that these genomes carried a large number of transposons and insertion sequences containing antibiotic-resistance genes, and Type I integrase genes were discovered in some composite transposons carrying antibiotic-resistance genes. The Tn4352 complex transposon in plasmid 1 of strain 11 carried both an integrase gene and the aminoglycoside-resistance gene *APH(3′)-Ia*. One complex transposon containing a Type I integrase gene was found in both plasmid 1 and plasmid 2 of strain 24, and the complex transposon in plasmid 1 contained the chloramphenicol efflux pump protein gene *cmlA6*, the ADP-ribosyltransferase gene *arr-2*, and the dihydrofolate reductase gene *dfrA14*. Furthermore, this composite transposon overlapped with the *cmlA6* gene in a Tn3 composite transposon carrying the florfeniol-resistance gene *floR*. In the composite transposon of plasmid 2, in addition to the Type I integrase and *aadA2* and *dfrA12* resistance genes, a gene island was observed containing the resistance genes *mphA* and *sul1*, although the Type I integrase gene was not located within the gene island. Unlike strains 11 and 24, strain 23 carried the Type I integrase gene in the gene island of the chromosome, which contained the *dfrA17*, *aadA5*, and *sul1* resistance genes, and its gene structure was more similar to that of the gene island in plasmid 2 of strain 24. Based on the above results and integrase structural analysis, it was inferred that the composite integrase on the plasmids and chromosomal gene islands contained Type I integrase, with some of these integrases carrying the *dfrA-aadA* resistance gene cassette. Due to the specific structure of the integrons’ multi-gene cassettes, the gene cassettes can be inserted at specific sites under the action of integrase, and the unique structure allows the integrons to capture a large number of exogenous mobile drug resistance genes and then to express these captured exogenous genes. At the same time, the integrons can be carried by transposons, gene islands, and other mobile elements, thus contributing to the horizontal transfer of drug-resistant genes, resulting in the intensification of bacterial drug resistance.

## 5 Conclusion

In this study, the isolation and identification of *E. coli* isolates from calf diarrhea from the Tongliao region, together with analysis of drug resistance, revealed that *E. coli* in this region is severely resistant to drugs, with 100% resistance to sulfadiazine sodium, enrofloxacin and ciprofloxacin. The detection rates of the antibiotic-resistance genes *TEM-1*, *TEM-206*, *strA*, *strB*, *qacH*, and *blaCTX* were 100%. Whole-genome sequencing of the three multi-drug resistant *E. coli* strains demonstrated that all three strains carried plasmids containing resistance genes. These resistance genes and MGE prediction showed a large number of transposons and insertion sequences in these genes in the strains. Furthermore, the presence of integrase genes was found in the plasmids and chromosomal genomes of several strains, contributing significantly to the horizontal transfer of drug-resistance genes, and thus leading to significantly increased resistance against antimicrobial drugs.

## Data Availability

The datasets presented in this study can be found in online repositories. The names of the repository/repositories and accession number(s) can be found at: https://www.ncbi.nlm.nih.gov/genbank/, CP157955-CP157957, CP157958-CP157959, CP103295-CP103297.
