# Supplementary figures and images for: Detection of drug resistance in Escherichia coli from calves with diarrhea in the Tongliao region: an analysis of multidrug-resistant strains
Source: Front Vet Sci. 2024 Nov 13;11:1466690. doi: 10.3389/fvets.2024.1466690 (PMC11601152; doi:10.3389/fvets.2024.1466690)

## Slide 1
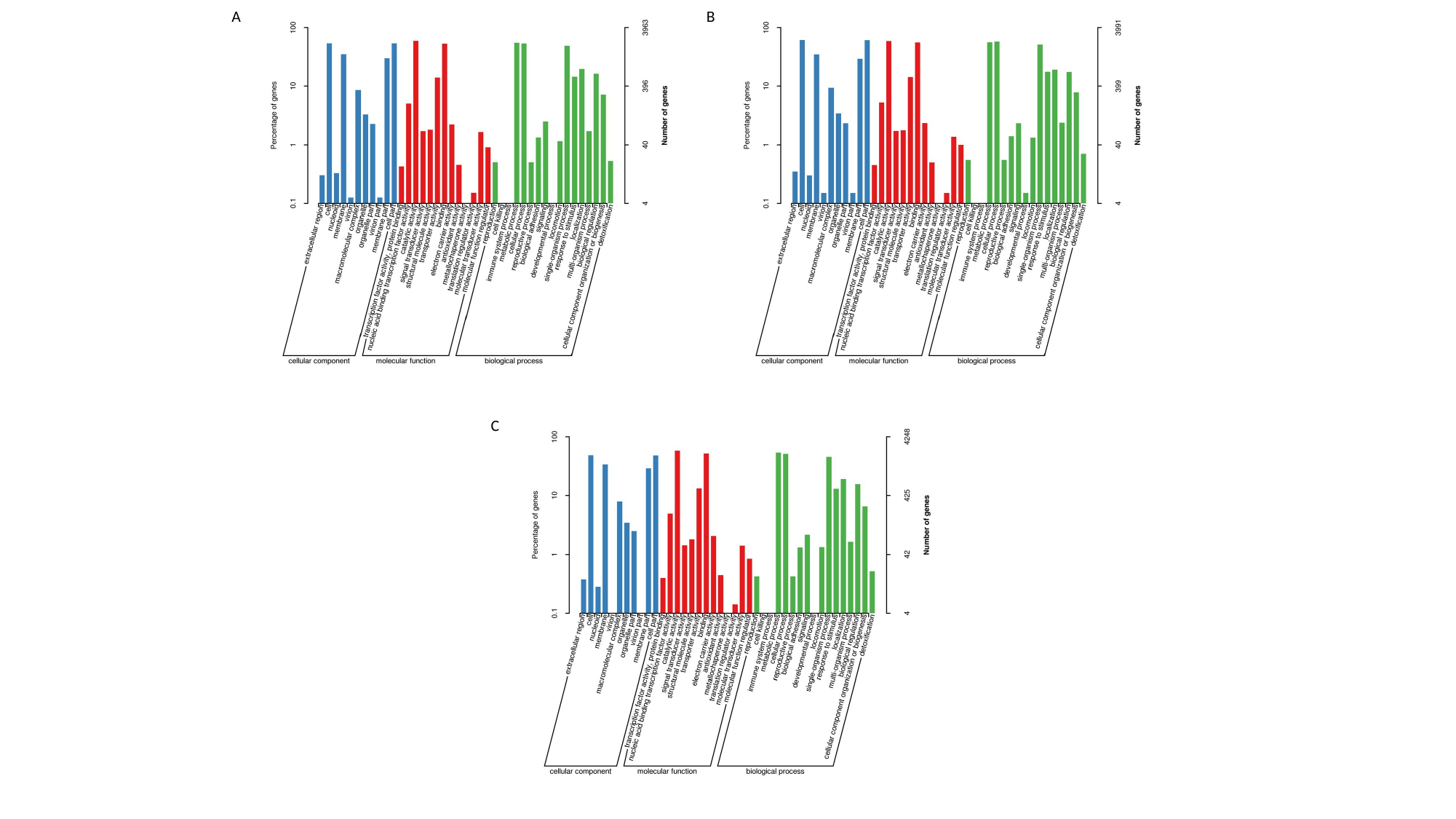

A
B
C

## Slide 2
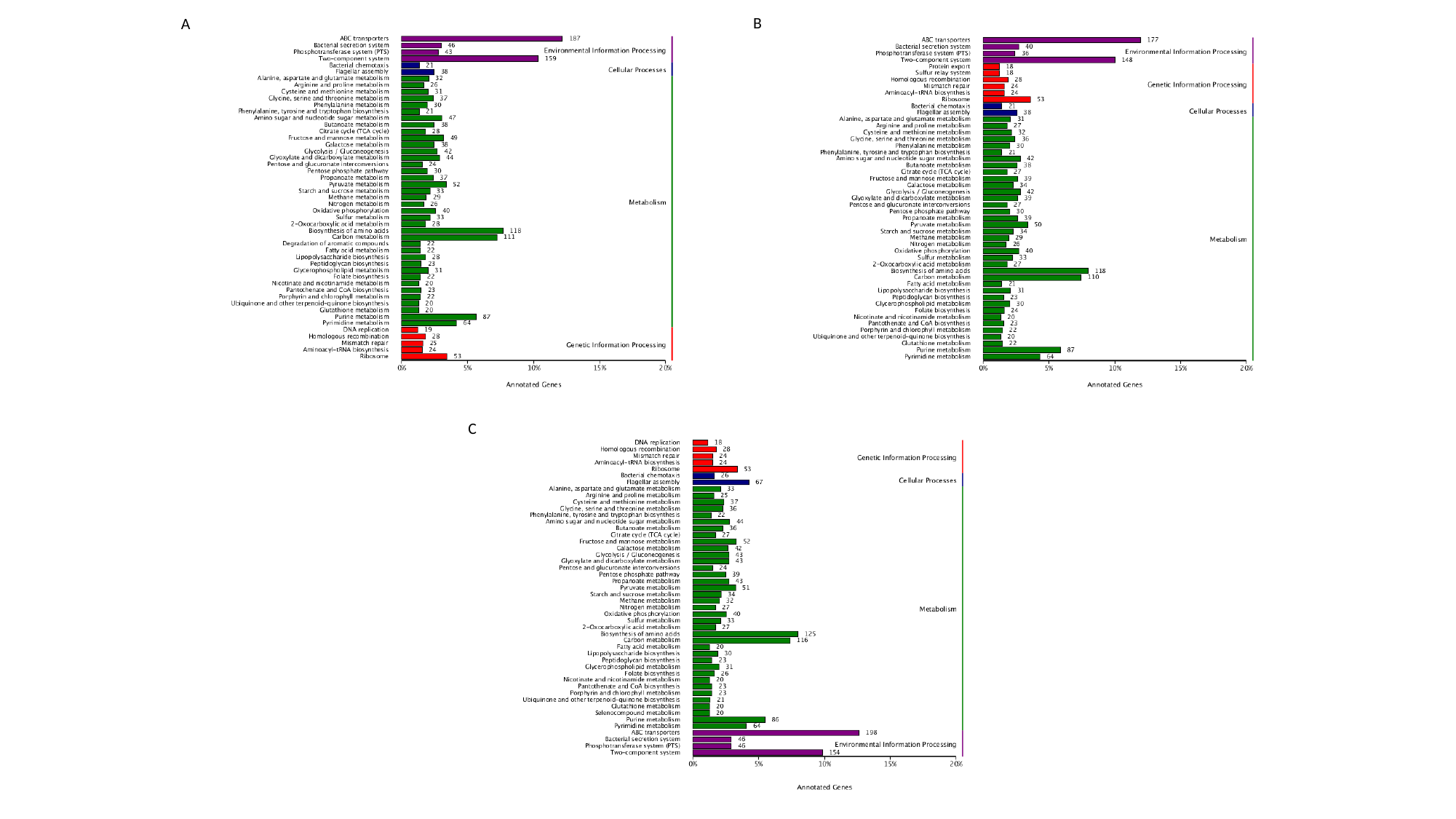

B
A
C

Supplement: Supplementary file 1 [file Presentation_1.pptx]

## Slide 1
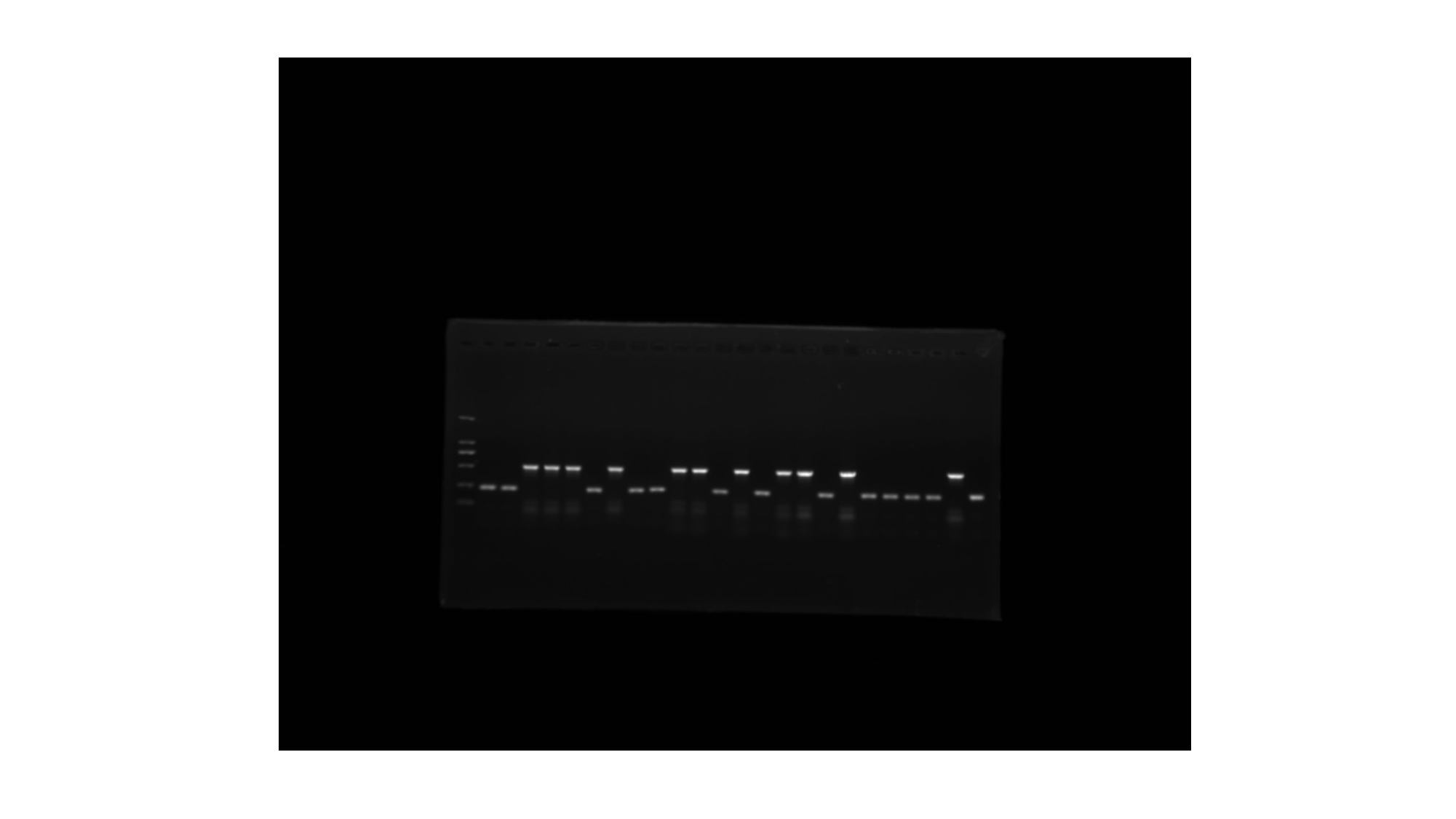

## Slide 2
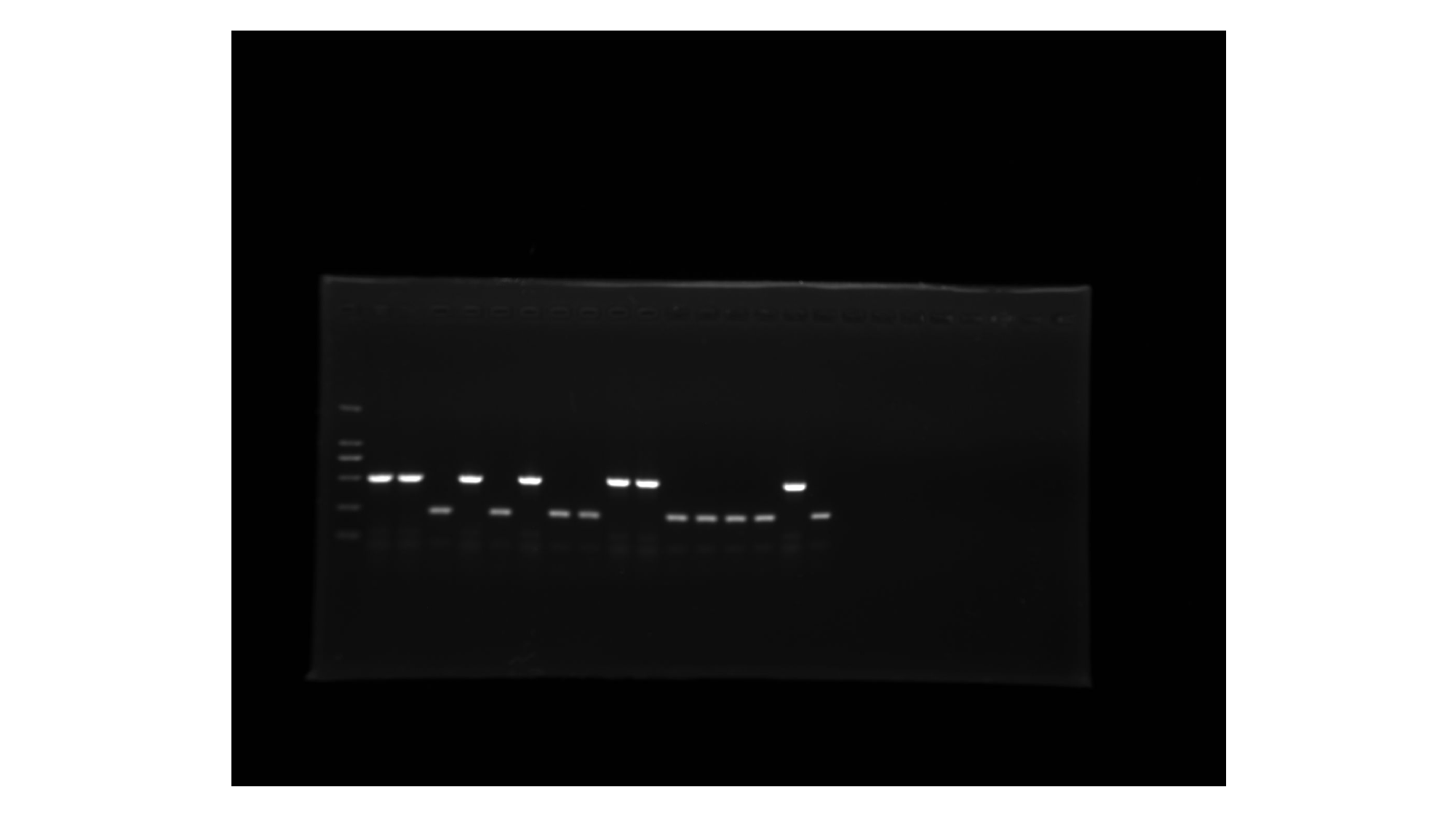

Supplement: Supplementary file 2 [file Presentation_2.pptx]
