# Supplementary material for: Detection of drug resistance in Escherichia coli from calves with diarrhea in the Tongliao region: an analysis of multidrug-resistant strains
Source: Front Vet Sci. 2024 Nov 13;11:1466690. doi: 10.3389/fvets.2024.1466690 (PMC11601152; doi:10.3389/fvets.2024.1466690)
Supplement: Supplementary file 3 [file Table_1.DOCX]

Table S1 Primer sequences of drug-resistance genes

| Primer name | Primer sequence (5´→3´) | The length of the amplification/bp |  | Primer name | Primer sequence (5´→3´) | The length of the amplification/bp |
| --- | --- | --- | --- | --- | --- | --- |
| aphA1 Pm-fw | AAAGCCGTTTCTGTAATGAAGGAG | 642 |  | TEM-1,F | TTTTCGTGTCGCCCTTAT | 512 |
| aphA1 Pm-rv | GGCAATCAGGTGCGACAATCT |  |  | TEM-1,R | GCTCGTCGTTTGGTATGG |  |
| strB Pm-fw | GCGTTGCTCCTCTTCTCCAT | 723 |  | TEM-206, F | TGAATGAAGCCATACCAA | 279 |
| strB Pm-rv | ACCTTTTCCAGCCTCGTTTG |  |  | TEM-206,R | AGATAACTACGATACGGGAG |  |
| strA Pm-fw | ATCGCAGATAGAAGGCAAGGC | 574 |  | blaCTX,F | CGCTTTGCGATGTGCAG | 550 |
| strA Pm-rv | AACTGGCAGGAGGAACAGGA |  |  | blaCTX,R | ACCGCGATATCGTTGGT |  |
| aadA25 Pm-fw | ACTATCAGAGGTGCTAAGCGTCAT | 724 |  | CTX-M-55,F | GCTGGGGTAAAGCATTGGGG | 224 |
| aadA25 Pm-rv | CACGTAGTGAACAAATTCTTCCAAC |  |  | CTX-M-55,R | TAGGTTGAGGCTGGGGTGA |  |
| aadA17, F | ATTCTTGCGGGGTATCTTCG | 309 |  | AAC(3)-Iia,F | TCACGAACTCGGTCACGC | 177 |
| aadA17,R | CTGGGCAGGTAGGCGTTT |  |  | AAC(3)-Iia,R | TCACGAACTCGGTCACGC |  |
| aadA5,F | CTTCAGTTCGGTGAGTGGC | 398 |  | qacH,F | TCGCACTCAAGTCCATCC | 150 |
| aadA5,R | AGGTCGTCGTCCTCGCTAC |  |  | qacH,R | CGACGCCACTAACGATAAG |  |
| aadA2,F | CATCCCGTGGCGTTATCC | 370 |  | Int1-F | CCTCCCGCACGATGATC | 280 |
| aadA2,R | CTGGGCAGGTAGGCGTTT |  |  | Int1-R | TCCACGCATCGTCAGGC |  |
| aadA,F | ATCTGGCTATCTTGCTGACA | 388 |  | catI,F | CAATGAAAGACGGTGAGC | 362 |
| aadA,R | TTGGTGATCTCGCCTTTC |  |  | catI,R | ACAAACGGCATGATGAAC |  |
| tetA, F | GCTACATCCTGCTTGCCTTC | 210 |  | cmlA6,F | GTACGACAGCGAGCACAA | 124 |
| tetA, R | CATAGATCGCCGTGAAGAGAGG |  |  | cmlA6,R | CCACCTCCCAGTAGAACG |  |
| tetD,F | ACAGCATTCTCGCTATCA | 146 |  | floR,F | CTGAACACGACGCCCGCTAT | 751 |
| tetD,R | TATTCGCCACATCATCAA |  |  | floR,R | GGACCGCTCCGCAAACAA |  |
| tetR,F | AACAACCCGTAAACTCGC | 116 |  | mprF,F | GGATGCAGGTTTGGTCTG | 282 |
| tetR,R | GGTGCCTATCTAACATCTCAAT |  |  | mprF,R | TCGTCAATACGGTTCTCACT |  |
| sul1,F | TTTCCTGACCCTGCGCTCTAT | 425 |  | qnrS-F | ACGACATTCGTCAACTGCAA | 417 |
| sul1,R | GTGCGGACGTAGTCAGCGCCA |  |  | qnrS-R | TAAATTGGCACCCTGTAGGC |  |
| sul2, F | CCTGTTTCGTCCGACACAGA | 435 |  |  |  |  |
| sul2, R | GAAGCGCAGCCGCAATTCAT |  |  |  |  |  |

Table S2 Drug resistance genes carried by strain 11

| Position | ARO-name | Gene-list |  | Position | ARO-name | Gene-list |
| --- | --- | --- | --- | --- | --- | --- |
| Chromosomal genomes | mdtE | GE000076 |  | Chromosomal genomes | kdpE | GE001599 |
| Chromosomal genomes | mdtF | GE000077 |  | Chromosomal genomes | Escherichia coli mdfA | GE001786 |
| Chromosomal genomes | gadX | GE000080 |  | Chromosomal genomes | Escherichia coli nfsA mutations conferring resistance to nitrofurantoin | GE001812 |
| Chromosomal genomes | cpxA | GE000474 |  | Chromosomal genomes | msbA | GE001874 |
| Chromosomal genomes | Escherichia coli EF-Tu mutants conferring resistance to Pulvomycin | GE000541 |  | Chromosomal genomes | mdtG | GE002009 |
| Chromosomal genomes | Escherichia coli soxS with mutation conferring antibiotic resistance | GE000637 |  | Chromosomal genomes | mdtH | GE002021 |
| Chromosomal genomes | Escherichia coli soxR with mutation conferring antibiotic resistance | GE000638 |  | Chromosomal genomes | H-NS | GE002188 |
| Chromosomal genomes | mdtP | GE000656 |  | Chromosomal genomes | Escherichia coli marR mutant conferring antibiotic resistance | GE002468 |
| Chromosomal genomes | mdtO | GE000657 |  | Chromosomal genomes | marA | GE002469 |
| Chromosomal genomes | mdtN | GE000658 |  | Chromosomal genomes | ugd | GE002993 |
| Chromosomal genomes | eptA | GE000691 |  | Chromosomal genomes | mdtA | GE003037 |
| Chromosomal genomes | Escherichia coli ampC beta-lactamase | GE000730 |  | Chromosomal genomes | mdtB | GE003038 |
| Chromosomal genomes | mdtM | GE000911 |  | Chromosomal genomes | mdtC | GE003039 |
| Chromosomal genomes | Haemophilus influenzae PBP3 conferring resistance to beta-lactam antibiotics | GE001060 |  | Chromosomal genomes | baeS | GE003041 |
| Chromosomal genomes | acrB | GE001387 |  | Chromosomal genomes | baeR | GE003042 |
| Chromosomal genomes | Escherichia coli acrA | GE001388 |  | Chromosomal genomes | YojI | GE003171 |
| Chromosomal genomes | Escherichia coli acrR with mutation conferring multidrug antibiotic resistance | GE001389 |  | Chromosomal genomes | Escherichia coli gyrA conferring resistance to fluoroquinolones | GE003190 |
| Chromosomal genomes | Escherichia coli GlpT with mutation conferring resistance to fosfomycin | GE003203 |  | Plasmid 1 genomes | TEM-1 | GE004415 |
| Chromosomal genomes | PmrF | GE003218 |  | Plasmid 1 genomes | mef(B) | GE004417 |
| Chromosomal genomes | emrY | GE003317 |  | Plasmid 1 genomes | sul3 | GE004422 |
| Chromosomal genomes | emrK | GE003318 |  | Plasmid 1 genomes | qacH | GE004424 |
| Chromosomal genomes | evgA | GE003319 |  | Plasmid 1 genomes | aadA | GE004425 |
| Chromosomal genomes | evgS | GE003320 |  | Plasmid 1 genomes | cmlA6 | GE004426 |
| Chromosomal genomes | evgS | GE003323 |  | Plasmid 1 genomes | aadA2 | GE004427 |
| Chromosomal genomes | acrD | GE003408 |  | Plasmid 1 genomes | dfrA12 | GE004428 |
| Chromosomal genomes | emrR | GE003597 |  | Plasmid 1 genomes | APH(3')-Ia | GE004431 |
| Chromosomal genomes | emrA | GE003598 |  | Plasmid 2 genomes | FosA3 | GE004520 |
| Chromosomal genomes | emrB | GE003599 |  | Plasmid 2 genomes | TEM-206 | GE004522 |
| Chromosomal genomes | Escherichia coli parC conferring resistance to fluoroquinolone | GE003954 |  | Plasmid 2 genomes | CTX-M-55 | GE004524 |
| Chromosomal genomes | TolC | GE003972 |  | Plasmid 2 genomes | TEM-1 | GE004547 |
| Chromosomal genomes | bacA | GE003994 |  | Plasmid 2 genomes | rmtB | GE004548 |
| Chromosomal genomes | AcrS | GE004192 |  | Plasmid 2 genomes | floR | GE004555 |
| Chromosomal genomes | AcrE | GE004193 |  | Plasmid 2 genomes | tet(C) | GE004558 |
| Chromosomal genomes | AcrF | GE004194 |  | Plasmid 2 genomes | APH(6)-Id | GE004561 |
| Chromosomal genomes | Escherichia coli EF-Tu mutants conferring resistance to Pulvomycin | GE004263 |  | Plasmid 2 genomes | vgaC | GE004572 |
| Chromosomal genomes | CRP | GE004282 |  |  |  |  |

Table S3 Drug resistance genes carried by strain 23

| Position | ARO-name | Gene-list |  | Position | ARO-name | Gene-list |
| --- | --- | --- | --- | --- | --- | --- |
| Chromosomal genomes | Haemophilus influenzae PBP3 conferring resistance to beta-lactam antibiotics | GE000079 |  | Chromosomal genomes | TEM-1 | GE001181 |
| Chromosomal genomes | acrB | GE000471 |  | Chromosomal genomes | APH(6)-Id | GE001183 |
| Chromosomal genomes | Escherichia coli acrA | GE000472 |  | Chromosomal genomes | APH(3'')-Ib | GE001184 |
| Chromosomal genomes | Escherichia coli acrR with mutation conferring multidrug antibiotic resistance | GE000473 |  | Chromosomal genomes | sul2 | GE001185 |
| Chromosomal genomes | kdpE | GE000685 |  | Chromosomal genomes | TEM-1 | GE001189 |
| Chromosomal genomes | Escherichia coli mdfA | GE000830 |  | Chromosomal genomes | sul2 | GE001194 |
| Chromosomal genomes | Escherichia coli nfsA mutations conferring resistance to nitrofurantoin | GE000860 |  | Chromosomal genomes | APH(3'')-Ib | GE001195 |
| Chromosomal genomes | msbA | GE000922 |  | Chromosomal genomes | APH(6)-Id | GE001196 |
| Chromosomal genomes | mdtG | GE001058 |  | Chromosomal genomes | H-NS | GE001279 |
| Chromosomal genomes | mdtH | GE001070 |  | Chromosomal genomes | Escherichia coli marR mutant conferring antibiotic resistance | GE001556 |
| Chromosomal genomes | tet(D) | GE001153 |  | Chromosomal genomes | marA | GE001557 |
| Chromosomal genomes | tet(A) | GE001155 |  | Chromosomal genomes | mdtA | GE002102 |
| Chromosomal genomes | tetR | GE001156 |  | Chromosomal genomes | mdtB | GE002103 |
| Chromosomal genomes | catI | GE001162 |  | Chromosomal genomes | mdtC | GE002104 |
| Chromosomal genomes | dfrA17 | GE001168 |  | Chromosomal genomes | baeS | GE002106 |
| Chromosomal genomes | aadA5 | GE001169 |  | Chromosomal genomes | baeR | GE002107 |
| Chromosomal genomes | sul1 | GE001171 |  | Chromosomal genomes | YojI | GE002234 |
| Chromosomal genomes | mphA | GE001178 |  | Chromosomal genomes | Escherichia coli gyrA conferring resistance to fluoroquinolones | GE002253 |
| Chromosomal genomes | Escherichia coli GlpT with mutation conferring resistance to fosfomycin | GE002262 |  | Chromosomal genomes | mdtE | GE003467 |
| Chromosomal genomes | PmrF | GE002280 |  | Chromosomal genomes | mdtF | GE003468 |
| Chromosomal genomes | emrY | GE002376 |  | Chromosomal genomes | gadW | GE003469 |
| Chromosomal genomes | emrK | GE002377 |  | Chromosomal genomes | gadX | GE003470 |
| Chromosomal genomes | evgA | GE002378 |  | Chromosomal genomes | tetR | GE003625 |
| Chromosomal genomes | evgS | GE002379 |  | Chromosomal genomes | tet(A) | GE003626 |
| Chromosomal genomes | acrD | GE002463 |  | Chromosomal genomes | tet(D) | GE003628 |
| Chromosomal genomes | emrR | GE002660 |  | Chromosomal genomes | cpxA | GE003931 |
| Chromosomal genomes | emrA | GE002661 |  | Chromosomal genomes | Escherichia coli EF-Tu mutants conferring resistance to Pulvomycin | GE004032 |
| Chromosomal genomes | emrB | GE002662 |  | Chromosomal genomes | Escherichia coli soxS with mutation conferring antibiotic resistance | GE004126 |
| Chromosomal genomes | Escherichia coli parC conferring resistance to fluoroquinolone | GE002987 |  | Chromosomal genomes | Escherichia coli soxR with mutation conferring antibiotic resistance | GE004127 |
| Chromosomal genomes | TolC | GE003002 |  | Chromosomal genomes | mdtP | GE004145 |
| Chromosomal genomes | bacA | GE003022 |  | Chromosomal genomes | mdtO | GE004146 |
| Chromosomal genomes | AcrS | GE003221 |  | Chromosomal genomes | mdtN | GE004147 |
| Chromosomal genomes | AcrS | GE003222 |  | Chromosomal genomes | eptA | GE004180 |
| Chromosomal genomes | AcrE | GE003223 |  | Chromosomal genomes | Escherichia coli ampC beta-lactamase | GE004218 |
| Chromosomal genomes | AcrF | GE003224 |  | Chromosomal genomes | mdtM | GE004414 |
| Chromosomal genomes | Escherichia coli EF-Tu mutants conferring resistance to Pulvomycin | GE003294 |  | Plasmid 1 genomes | APH(6)-Id | GE004584 |
| Chromosomal genomes | CRP | GE003313 |  | Plasmid 1 genomes | APH(3'')-Ib | GE004585 |
| Plasmid 1 genomes | tetR | GE004593 |  | Plasmid 1 genomes | tet(D) | GE004596 |
| Plasmid 1 genomes | tet(A) | GE004594 |  | Plasmid 1 genomes | TEM-1 | GE004600 |

Table S4 Drug resistance genes carried by strain 24

| Position | ARO-name | Gene-list |  | Position | ARO-name | Gene-list |
| --- | --- | --- | --- | --- | --- | --- |
| Chromosomal genomes | gadX | GE000222 |  | Chromosomal genomes | emrY | GE001484 |
| Chromosomal genomes | gadW | GE000223 |  | Chromosomal genomes | PmrF | GE001581 |
| Chromosomal genomes | mdtF | GE000225 |  | Chromosomal genomes | Escherichia coli GlpT with mutation conferring resistance to fosfomycin | GE001596 |
| Chromosomal genomes | mdtE | GE000226 |  | Chromosomal genomes | Escherichia coli gyrA conferring resistance to fluoroquinolones | GE001609 |
| Chromosomal genomes | CRP | GE000383 |  | Chromosomal genomes | YojI | GE001628 |
| Chromosomal genomes | Escherichia coli EF-Tu mutants conferring resistance to Pulvomycin | GE000402 |  | Chromosomal genomes | baeR | GE001755 |
| Chromosomal genomes | AcrF | GE000457 |  | Chromosomal genomes | baeS | GE001756 |
| Chromosomal genomes | AcrE | GE000458 |  | Chromosomal genomes | mdtC | GE001758 |
| Chromosomal genomes | AcrS | GE000459 |  | Chromosomal genomes | mdtB | GE001759 |
| Chromosomal genomes | bacA | GE000661 |  | Chromosomal genomes | mdtA | GE001760 |
| Chromosomal genomes | TolC | GE000680 |  | Chromosomal genomes | ugd | GE001802 |
| Chromosomal genomes | Escherichia coli parC conferring resistance to fluoroquinolone | GE000700 |  | Chromosomal genomes | marA | GE002262 |
| Chromosomal genomes | emrB | GE001135 |  | Chromosomal genomes | Escherichia coli marR mutant conferring antibiotic resistance | GE002263 |
| Chromosomal genomes | emrA | GE001136 |  | Chromosomal genomes | H-NS | GE002588 |
| Chromosomal genomes | emrR | GE001137 |  | Chromosomal genomes | mdtH | GE002770 |
| Chromosomal genomes | acrD | GE001394 |  | Chromosomal genomes | mdtG | GE002783 |
| Chromosomal genomes | evgS | GE001481 |  | Chromosomal genomes | CMY-60 | GE002836 |
| Chromosomal genomes | evgA | GE001482 |  | Chromosomal genomes | CMY-43 | GE002839 |
| Chromosomal genomes | emrK | GE001483 |  | Chromosomal genomes | CMY-2 | GE002865 |
| Chromosomal genomes | msbA | GE003000 |  | Chromosomal genomes | cpxA | GE004479 |
| Chromosomal genomes | Escherichia coli nfsA mutations conferring resistance to nitrofurantoin | GE003064 |  | Chromosomal genomes | Escherichia coli CyaA with mutation conferring resistance to fosfomycin | GE004600 |
| Chromosomal genomes | Escherichia coli mdfA | GE003073 |  | Plasmid 1 genomes | Brucella suis mprF | GE004734 |
| Chromosomal genomes | kdpE | GE003211 |  | Plasmid 1 genomes | CTX-M-55 | GE004790 |
| Chromosomal genomes | Escherichia coli acrR with mutation conferring multidrug antibiotic resistance | GE003413 |  | Plasmid 1 genomes | APH(3')-Ia | GE004899 |
| Chromosomal genomes | Escherichia coli acrA | GE003414 |  | Plasmid 1 genomes | QnrS1 | GE004915 |
| Chromosomal genomes | acrB | GE003415 |  | Plasmid 1 genomes | aadA17 | GE004922 |
| Chromosomal genomes | Haemophilus influenzae PBP3 conferring resistance to beta-lactam antibiotics | GE003836 |  | Plasmid 1 genomes | linG | GE004923 |
| Chromosomal genomes | mdtM | GE003980 |  | Plasmid 1 genomes | AAC(3)-IIa | GE004926 |
| Chromosomal genomes | Escherichia coli ampC  beta-lactamase | GE004174 |  | Plasmid 1 genomes | TEM-1 | GE004931 |
| Chromosomal genomes | floR | GE004202 |  | Plasmid 1 genomes | APH(6)-Id | GE004932 |
| Chromosomal genomes | sul2 | GE004206 |  | Plasmid 1 genomes | APH(3'')-Ib | GE004933 |
| Chromosomal genomes | eptA | GE004249 |  | Plasmid 1 genomes | sul2 | GE004934 |
| Chromosomal genomes | mdtN | GE004282 |  | Plasmid 1 genomes | floR | GE004960 |
| Chromosomal genomes | mdtO | GE004283 |  | Plasmid 1 genomes | cmlA6 | GE004967 |
| Chromosomal genomes | mdtP | GE004284 |  | Plasmid 1 genomes | arr-2 | GE004968 |
| Chromosomal genomes | Escherichia coli soxR with mutation conferring antibiotic resistance | GE004301 |  | Plasmid 1 genomes | dfrA14 | GE004969 |
| Chromosomal genomes | Escherichia coli soxS with mutation conferring antibiotic resistance | GE004302 |  | Plasmid 2 genomes | tet(C) | GE005036 |
| Chromosomal genomes | Escherichia coli EF-Tu mutants conferring resistance to Pulvomycin | GE004409 |  | Plasmid 2 genomes | TEM-1 | GE005038 |
| Plasmid 2 genomes | AAC(3)-IIa | GE005042 |  | Plasmid 2 genomes | aadA23 | GE005070 |
| Plasmid 2 genomes | TEM-1 | GE005047 |  | Plasmid 2 genomes | tet(C) | GE005076 |
| Plasmid 2 genomes | sul2 | GE005060 |  | Plasmid 2 genomes | mphA | GE005105 |
| Plasmid 2 genomes | APH(3'')-Ib | GE005061 |  | Plasmid 2 genomes | sul1 | GE005112 |
| Plasmid 2 genomes | APH(6)-Id | GE005062 |  | Plasmid 2 genomes | aadA2 | GE005114 |
| Plasmid 2 genomes | APH(3')-Ia | GE005063 |  | Plasmid 2 genomes | dfrA12 | GE005115 |

Table S5 Mobile elements carrying drug resistance genes in strain 11

| Position | Composite transposon | Insertion sequence | Family | Strand | Resistance gene |
| --- | --- | --- | --- | --- | --- |
| Plasmid 1 genomes | Composite transposon | No | None | forward | APH(3’)-Ia |
| Plasmid 2 genomes | Composite transposon | No | IS6 | reverse | TEM-1、rmtB |
| Plasmid 2 genomes | Composite transposon | No | IS6 | forward | FosA3 |

Table S6 Mobile elements carrying drug resistance genes in strain 23

| Position | Composite transposon/Unit transposon | Insertion sequence | Family | Strand | Resistance gene |
| --- | --- | --- | --- | --- | --- |
| Chromosomal genomes | Composite transposon | No | IS4 | reverse | tet(B) |
| Plasmid 1 genomes | Unit transposon | No | None | forward | TEM-1 |
| Plasmid 1 genomes | Composite transposon | No | IS4 | forward | tet(B) |

Table S7 Mobile elements carrying drug resistance genes in strain 24

| Position | Composite transposon | Insertion sequence | Family | Strand | Resistance gene |
| --- | --- | --- | --- | --- | --- |
| Chromosomal genomes | Composite transposon | No | IS91 | reverse | floR |
| Chromosomal genomes | Composite transposon | No | IS1380 | reverse | blaCMY-2 |
| Plasmid 1 genomes | Composite transposon | No | IS6 | forward | qnrS-1 |
| Plasmid 1 genomes | Composite transposon | Yes | IS6 | reverse | cmlA6、arr-2、dfrA14、floR |
| Plasmid 1 genomes | Composite transposon | Yes | IS66 | reverse | mprF |
| Plasmid 1 genomes | Composite transposon | No | IS6 | forward | APH(3’)-Ia |
| Plasmid 2 genomes | Composite transposon | No | IS6 | reverse | AAC(3)-IIa、TEM-1 |
| Plasmid 2 genomes | Composite transposon | No | IS6 | forward | sul2、APH(3’)-Ib、APH(6)-Id、APH(3’)-Ia |
| Plasmid 2 genomes | Composite transposon | No | IS6 | reverse | mphA、qacE、sul1、aadA2、dfrA12 |
